# Application and Challenges of Using Probiotic *Lactobacillus* and *Bifidobacterium* to Enhance Overall Health and Manage Diseases

**DOI:** 10.3390/diseases13100345

**Published:** 2025-10-17

**Authors:** Kawaljit Kaur

**Affiliations:** ImmuneLink, LLC, Riverside, CA 92508, USA; drkawalmann@g.ucla.edu; Tel.: +1-5093395967

**Keywords:** NK cells, T cells, CD8+ T cells, immunotherapy, probiotics, cancer, aging, autoimmune, neurodegenerative, cytotoxicity, IFN-γ

## Abstract

**Simple Summary:**

Probiotics are live microorganisms that support health by balancing gut bacteria, boosting immunity and metabolism, and potentially preventing or treating various conditions. Their effectiveness varies by strain and proper use. Advances in microbiome research are leading to personalized probiotic treatments with significant benefits. Different strains play unique roles in aging, cancer, autoimmune diseases, and neurodegenerative disorders. Understanding these strain-specific effects is crucial for personalized healthcare. Studies show probiotics can modulate immunity, gut health, and overall well-being. Tailoring probiotics to individual needs may help address complex health challenges. This review provides an overview of probiotic strains *Lactobacillus* and *Bifidobacterium* mediated unique benefits, suggesting that targeted use can maximize their therapeutic potential.

**Abstract:**

Probiotics are known for their health benefits, and new studies suggest they could help with various conditions. However, the specific formulations and mechanisms of probiotics in addressing these issues are still being explored. This review focuses on four key areas: cancer, aging, autoimmune diseases, and neurodegenerative disorders, highlighting the potential benefits of *Lactobacillus* and *Bifidobacterium* probiotics. Their interaction with the immune system plays a crucial role in offering protection and therapeutic effects, particularly in enhancing immunity in older adults. The review sheds light on how these probiotics affect the immune system, gut microbiome, and related processes to manage or combat these health problems. It emphasizes the importance of customizing probiotic formulations for specific conditions, as different combinations of *Lactobacillus* and *Bifidobacterium* uniquely activate immune cells. Some combinations work as effective treatments for diseases, while others boost immunity in aging. While the potential of these probiotics is significant, challenges remain in using them for cancer, age-related diseases, autoimmune diseases neurodegenerative disorder treatments. Limited evidence calls for further research to define their role and establish guidelines. Future approaches like strain engineering, nanoencapsulation, synbiotics, and personalized microbiome analysis aim to overcome these challenges, making probiotics a more viable option for disease prevention and care. Additionally, there is an urgent need for clinical trials to ensure patients can benefit from these probiotics.

## 1. Introduction and Background

In the early 20th century, Elie Metchnikoff discovered certain strains of gut bacteria essential for maintaining balance in the gut, which he named probiotics [1]. These live, beneficial bacteria, often derived from fermented foods or the gut itself, offer numerous health benefits supported by studies and clinical trials [2,3]. Probiotics help maintain the intestinal barrier and microbial balance by enhancing proteins and mucus that prevent pathogens and excessive immune responses [4,5]. They interact with immune cells through direct contact and signaling molecules, boosting immunity while maintaining gut tolerance [6]. Probiotics also strengthen defenses against diseases and infections by activating immune cells and increasing cytokines and antibodies [7,8]. By competing with pathogens and producing antimicrobial agents, probiotics contribute to immune stability [9]. Components like exopolysaccharides, surface proteins, and secreted peptides interact with immune receptors, influencing signaling pathways [10,11,12]. They also adhere to the gut lining and engage with immune cells via Toll-like receptors (TLRs) like TLR2, TLR4, and TLR9 [8,13].

Probiotics play a crucial role in maintaining immune balance by activating regulatory T (Tregs) cells, which promote tolerance to commensals and food antigens, reducing inflammation and allergic responses [14]. They also impact the differentiation and function of T helper (Th) cell subsets and Tregs, driving immune responses like cell-mediated immunity or tolerance through cytokines like IL-10 and TGF-β [8,13]. Probiotics aid in B-cell maturation into plasma cells that produce secretory IgA (sIgA), essential for defending mucosal surfaces against pathogens [15]. The immune-modulating effects of probiotics depend heavily on the specific strains used, as different species and strains can uniquely affect cytokine responses and immune cell activity. For instance, *Lactobacillus* strains often enhance Th1-type immune responses, while *Bifidobacterium* strains typically promote anti-inflammatory effects [16]. Most probiotics are lactic acid-producing bacteria, such as lactobacilli, streptococci, and bifidobacteria, emphasizing their potential for human health [17]. Certain strains, like Lactococcus lactis subsp. Cremoris C60, have even been found to boost antigen presentation by dendritic cells (DCs), enhancing cytotoxic T cell responses critical for combating tumors and viruses [18,19].

*Lactobacillus* and *Bifidobacterium* offer promising benefits for cancer, aging, autoimmune diseases, and neurodegenerative disorders through their role in the gut–brain axis. However, their practical application faces hurdles like strain-specific variability, challenges with oral delivery and formulation stability, individual differences in gut microbiota, inconsistent clinical trial results, unclear mechanisms, disease-specific effectiveness, safety concerns for vulnerable groups, and environmental and lifestyle influences. Selecting the right strain and dosage is crucial for boosting immunity and maximizing health benefits. This review explores their mechanisms of action, applications, and challenges, emphasizing the importance of careful *Lactobacillus* and *Bifidobacterium* strain selection to achieve therapeutic outcomes. It highlights how specific strains enhance immune cell activity, maintain immune balance, and improve treatments for these conditions. Additionally, it stresses the need for faster clinical trials to bridge gaps between preclinical and clinical studies, ensuring patients can benefit from probiotics.

## 2. Mechanism of Action, Application, and Challenges of *Lactobacillus* and *Bifidobacterium* in Cancer Prevention and Their Role as Adjuvant Cancer Therapies

**Figure 1:**
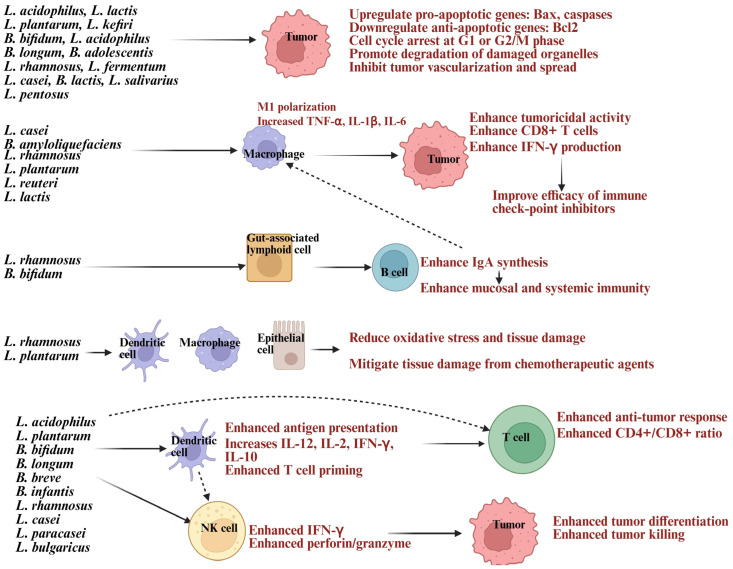
Illustration showing *Lactobacillus* and *Bifidobacterium* strains inducing direct apoptosis in tumor or modulating immune cells to enhance anti-cancer activity. Created in BioRender. Kaur, K. (2025) https://BioRender.com/a2qdrnd (accessed on 3 October 2025).

Cancer remains the leading cause of death worldwide, with cancer-related fatalities expected to increase [20,21,22]. There is a pressing demand for treatments that are both effective and have minimal side effects [23]. Current therapies often come with challenges like side effects, drug resistance, and affordability issues, which can affect quality of life [24]. Interestingly, studies show that probiotics may play a role in cancer prevention and work as complementary treatments [25]. Probiotics can prevent cancer by boosting immune function and enhancing the effectiveness of therapies. Even inactive probiotics and their byproducts provide similar benefits, making them a promising option for cancer prevention and treatment [26]. Among the probiotics being studied, this review focuses on *Lactobacillus* and *Bifidobacterium* strains as illustrated in Figure 1. *Lactobacillus* and *Bifidobacterium* species are recognized for their anti-cancer properties, achieved through direct toxicity to tumor cells and indirect effects on host physiology [27,28].

*Lactobacillus*, a prominent group of probiotic lactic acid bacteria (LAB), promotes gut health by inhibiting harmful pathogens like Fusobacterium nucleatum, Escherichia coli, and Clostridium difficile, which are linked to colorectal and other cancers [29,30]. *Bifidobacterium* combats cancer by inducing apoptosis, modulating oncogenic signaling pathways, activating the immune system, altering metabolism, and colonizing tumor hypoxic regions [31,32]. It also serves as a therapeutic agent and delivery vector for drugs, genes, and nanomaterials, offering a comprehensive approach to cancer prevention and treatment [33]. These probiotics induce apoptosis, growth cycle arrest, inhibit tumor vascularization, and metastasis [34]. *Lactobacillus* and *Bifidobacterium* produce antimicrobial substances, organic acids, bacteriocins, hydrogen peroxide, and short-chain fatty acids (SCFAs) like butyrate, acetate, and propionate, which reduce carcinogen-producing bacteria or trigger cancer cell apoptosis [35]. SCFAs regulate tumor suppressor genes and silence oncogenes through histone acetylation and methylation [36]. Exopolysaccharides (EPS) from *Lactobacillus plantarum* and *Lactobacillus acidophilus* activate TLR2/MyD88 pathways, promoting Fas-mediated apoptosis. They suppress tumor growth by inhibiting cyclin D1, causing G0/G1 phase arrest, and reducing cell proliferation [37,38]. EPS also activates pattern recognition receptors like TLR2, encouraging apoptosis, autophagy, and immune responses [11,37]. Bacteriocins and peptides damage tumor cell membranes, inducing apoptosis [39]. Probiotics induce cancer cell apoptosis by increasing pro-apoptotic proteins like Bax and BAD, decreasing anti-apoptotic proteins like Bcl-2, and activating caspase cascades, leading to deoxyribonucleic acid (DNA) fragmentation [40,41]. *Lactobacillus* and *Bifidobacterium* induced reduction in pro-angiogenic factors, further suppressing tumor growth, adding to on therapeutic value [42,43].

*Lactobacillus* and *Bifidobacterium* have anti-cancer properties by modulating the immune system. They enhance immune responses and tumor immunity by interacting with dendritic cells, macrophages, natural killer (NK) cells, and neutrophils, boosting their function and cytokine production [32]. They enhance the cytotoxic activity of innate immune cells like NK cells and CD8+ T cells, activate antigen-presenting cells such as DCs, and regulate cytokines by increasing interferon-gamma (IFN-γ) and IL-2 while reducing immunosuppressive IL-10 and TGF-β [44,45]. DCs are activated through CpG-rich bacterial DNA to promote Th1 responses via IL-12 signaling and IFN-γ production [44,45]. This leads to the upregulation of co-stimulatory molecules on antigen-presenting cells and the release of cytokines to manage inflammation and recruit immune cells, balancing pro- and anti-inflammatory pathways [19,46,47,48,49,50,51] (Figure 1). These probiotics influence macrophages, DCs, and intestinal epithelial cells, lowering the activity of inducible nitric oxide synthase (iNOS), which produces nitric oxide (NO) during inflammation [13,52]. By reducing iNOS levels, probiotics help prevent excessive NO production, minimizing oxidative stress and tissue damage [13]. They lower pro-inflammatory cytokines like TNF-α, IL-6, and IL-1β while increasing anti-inflammatory cytokines such as IL-10, maintaining balance to control chronic inflammation, strengthening the gut barrier, and regulating immune responses throughout the body [13,52]. In individuals consuming *Bifidobacterium*, DCs showed increased expression of anti-tumor immunity genes, boosting T cell activation [53]. These bacteria activate macrophages and aid in cancer prevention and treatment by influencing IgA production, stimulating macrophage activity, and reducing the toxicity of anti-cancer therapies [44,45,54]. These probiotics also promote macrophage polarization to an M1 anti-tumor phenotype, enhancing phagocytosis and cytokine-driven tumor suppression [44,45,54] (Figure 1).

**Figure 2:**
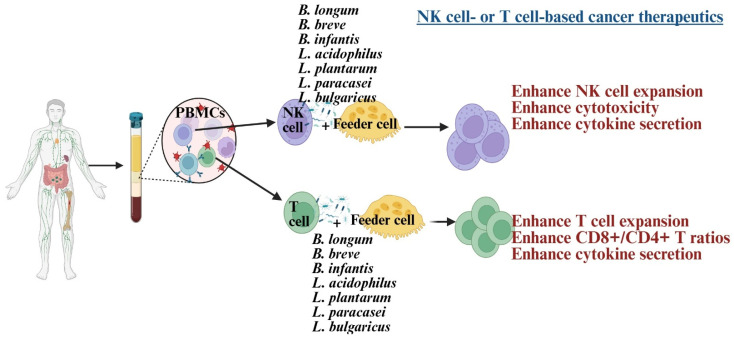
Illustration showing *Lactobacillus* and *Bifidobacterium* strains, when combined with feeder cells, induce cell expansion and enhance anti-cancer activity in NK cells and T cells. Created in BioRender. Kaur, K. (2025) https://BioRender.com/1smaneg (accessed on 3 October 2025).

NK cells and CD8+ T cells play a crucial role in cancer therapy, serving as the basis of many current treatments [32,55,56]. Dysfunction of these cells is linked to poorer outcomes in cancer patients [57]. In such patients, NK cells exhibit reduced cytotoxicity, IFN-γ secretion, survival, and expansion, associated with lower expression of CD16, NKG2D, and the CD3 zeta chain (CD3z) [58,59,60,61]. Similarly, CD8+ T cells show decreased IFN-γ secretion and reduced expression of CD62L, CD28, CCR7, and CD127 [62]. Probiotic bacteria have been explored to develop NK or CD8+ T cell-based immunotherapies, significantly enhancing these cells’ functions [58,60,62,63] (Figure 2). Combining probiotics with feeder cells has proven more effective for NK cell therapy than using feeder cells alone [58,62,64]. These expanded NK cells were more effective at inducing tumor killing and differentiation in both in vivo and in vitro studies [59,65]. The efficacy test of expanded T cells is still under investigation in tumor-bearing humanized mice.

Consuming *Lactobacillus acidophilus* is linked to increased serum levels of IFN-γ, IL-10, and higher counts of CD4+ and CD8+ T cells [66,67,68]. Oral administration of B. longum and B. breve to melanoma-bearing mice significantly reduced tumor volume, showing effects comparable to programmed death protein-1 (PD-1) therapy alone. When combined with PD-1 therapy, these probiotics further reduced tumor size [53]. *Lactobacillus* and *Bifidobacterium* probiotic-fed humanized mice showed improved NK-mediated cytotoxicity, increased IFN-γ secretion across tissues, reduced tumor load, and restored cancer-induced bone defects [59,60,65]. In mice with breast tumors, oral *Lactobacillus acidophilus* administration lowered tumor burden and influenced cytokine production [69]. *Bifidobacterium longum*, *Lactobacillus acidophilus*, and *Lactobacillus plantarum* have shown promise in preventing and inhibiting cancers such as colon, breast, liver, intestines, lungs, oral cavity, and pancreas [35,51,66,67,68,70,71,72,73,74,75,76,77,78].

These findings emphasize the potential of probiotics in cancer treatment, demonstrating their ability to modulate immune responses for therapeutic benefits [75,76,79]. When combined with chemotherapy and radiotherapy, probiotics enhance drug accumulation in tumors and reduce systemic side effects like diarrhea, fever, and disruptions to intestinal microbiota in cancer patients. These benefits improve patients’ quality of life and help prevent interruptions or halts in treatment [80]. Additionally, engineered *Bifidobacterium* can deliver drugs, cytokines, anti-angiogenic genes (such as endostatin or tumstatin), or prodrug-converting enzymes (like cytosine deaminase for converting 5-FC to 5-FU) directly to tumors, increasing local impact while minimizing systemic toxicity. They may also enhance focused ultrasound and imaging-based cancer therapies for precise targeting.

*Lactobacillus* and *Bifidobacterium* show potential in cancer care by improving gut microbiota, triggering cancer cell death, and boosting immune responses. However, their benefits face hurdles like strain-specific effects, individual variability, low survival rates, risks for immunocompromised individuals, formulation challenges, limited clinical evidence, and regulatory concerns, such as antibiotic resistance [32,81,82]. These probiotics may interact with other treatments, with effectiveness varying by individual and cancer type. Standardizing doses and formulations is crucial for safety and consistency [32,81,82]. Their anti-cancer effects are strain-dependent, influencing apoptosis, immune responses, and tumor environments, though some strains, like *Bifidobacterium dentium*, could be harmful [83]. Mechanisms include inducing tumor cell death and modulating the immune system, but the molecular pathways and tumor-specific impacts remain unclear [31]. Factors like dysbiosis, age, diet, genetics, gut microbiota, and health affect colonization and immune response [32,81,82]. Long-term colonization relies on adhesion mechanisms like exopolysaccharides or biofilms, supported by prebiotics. Probiotics face challenges from stomach acid, bile, and digestion, but encapsulation methods like alginate or chitosan enhance survival with strain-specific adjustments [32,81,82]. Effective doses depend on the strain and therapeutic goals, ranging from immune support to anti-proliferative effects [32,81,82]. Future advancements like strain engineering, nanoencapsulation, synbiotics, and personalized microbiome analysis aim to address these challenges, making probiotics a more viable option in cancer care and prevention [84].

## 3. Mechanism of Action, Application, and Challenges of Utilizing *Lactobacillus* and *Bifidobacterium* to Restore Immune Function in Older Individuals

Aging brings about immunosenescence, characterized by a decline in both innate and adaptive immune functions, increased systemic inflammation, and alterations in the composition and diversity of gut microbiota [85,86]. It significantly affects innate immunity, causing changes in the number, characteristics, and functionality of immune cells, which are closely linked to various diseases and infections [87,88,89,90]. Additionally, cytokine levels, especially IFN-γ, are notably lower in the immune cells of older individuals [91]. These changes increase susceptibility to infections, chronic diseases, and reduced vaccine effectiveness. In older adults, diminished immune function leads to higher risks of infections, cancer, autoimmune disorders, Alzheimer’s disease, atherosclerosis, vision problems like age-related macular degeneration, cardiovascular issues, coronary heart disease, liver fibrosis, neurodegenerative diseases, and exposure to pathogens [90,92,93]. The gut microbiota is essential for immune regulation, with aging-related dysbiosis playing a major role in immune dysfunction [94,95].

NK cells are vital for fighting age-related illnesses and activating adaptive immune cells, ensuring a robust immune system in old age [87,91]. NK cells are thought to significantly influence longevity, as their proper function protects the elderly from infections, cancer, autoimmune disorders, and neurodegenerative diseases [87,90,91]. Aging leads to decreased NK cell function, contributing to age-related health issues [90]. Additionally, NK cell proliferation declines in the elderly. Research by Guo et al. found that the pro-inflammatory CD52+ NK cell subset increases in older adults, facilitating infection spread [96]. Feeder cells, which support NK cell activation and growth, also lose effectiveness in older individuals due to reduced ligand expression and factor secretion [91]. Probiotics can restore gut microbial balance, enhance innate immunity, such as NK cells, and regulate inflammation [4,13,97]. Probiotic supplements have been found to moderately improve immune markers and boost NK cell function, an essential part of innate immunity, in healthy older adults [13]. Studies show that probiotics significantly enhance NK cell cytotoxic activity in individuals aged 60 and above, helping with early infection defense and tumor surveillance [98]. They also lower chronic low-grade inflammation, prevent infections, and promote healthy aging [98].

**Figure 3:**
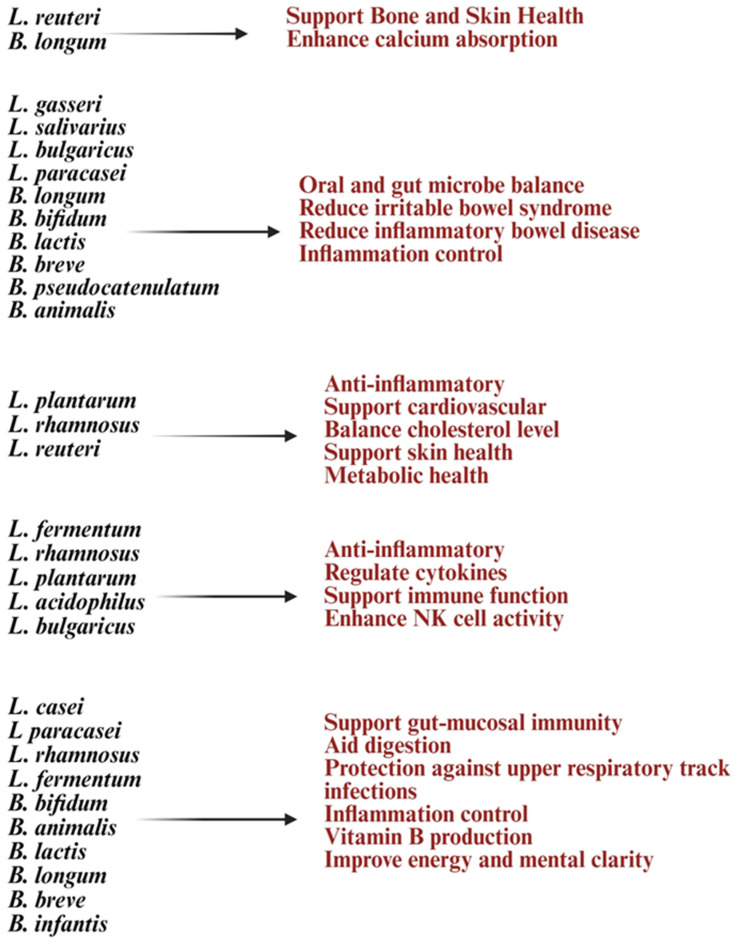
Illustration list of *Lactobacillus* and *Bifidobacterium* strains and their health benefits at old age. Created in BioRender. Kaur, K. (2025) https://BioRender.com/08dx43n (accessed on 3 October 2025).

Early studies suggest that strains like *Lactobacillus plantarum*, *Lactobacillus rhamnosus*, *Bifidobacterium longum*, and *Lactobacillus helveticus* may help improve aging processes and strengthen immunity [27]. *Lactobacillus* and *Bifidobacterium* increase anti-inflammatory cytokines like IL-10 and TGF-β while reducing pro-inflammatory markers such as IL-6 and CRP, combating chronic inflammation [8,99]. These treatments can activate T and B cells, improve immune monitoring, reduce the occurrence and severity of colds and gut infections, and strengthen vaccine responses [8,99]. Also, in vitro treatment with *Lactobacillus* and *Bifidobacterium* probiotics, alone or with feeder cells, has restored NK cell numbers and function in the elderly [91] (Figure 2). *Lactobacillus acidophilus* helps balance gut bacteria and boost immunity during illness or antibiotic use [9]. *Lactobacillus fermentum* strengthens the immune system and helps prevent gastrointestinal and respiratory infections [30,100]. *Lactobacillus casei* and *Lactobacillus paracasei* have anti-inflammatory properties, support the gut barrier, and aid in immune modulation [101]. *Lactobacillus plantarum* promotes immune response and reduces gut inflammation [102]. *Lactobacillus rhamnosus* enhances gut health, strengthens immune defenses, and eases digestive discomfort. *Bifidobacterium longum* reduces inflammation, protects against intestinal infections, and supports immune balance [103]. *Bifidobacterium bifidum* improves digestive health and boosts the immune system. *Bifidobacterium lactis* helps prevent infections and supports vitamins such as vitamin B and vitamin K production [83]. These vitamins are vital for metabolism and immune health. Probiotic formulations can be single-strain or multi-strain combinations, often including both *Lactobacillus* and *Bifidobacterium* species for synergistic benefits (Figure 3). Probiotics are generally safe for healthy elderly individuals [104].

*Lactobacillus* and *Bifidobacterium* improve antioxidant capacity in the gut and circulation, regulate microbial balance to maintain homeostasis, and may counteract age-related dysbiosis and illness [105,106]. Strains like *Lactobacillus plantarum* and *Lactobacillus reuteri* are linked to reduced oxidative stress and neuroinflammation [107]. They also support cognitive health and neuroprotection by modulating the gut–brain axis, benefiting memory, mood, and learning [108,109]. These probiotics influence neurotransmitters such as gamma-aminobutyric acid (GABA), serotonin, and dopamine, which help delay age-related cognitive decline and affect mood, stress, and cognition [110,111,112]. SCFAs and microbial metabolites reduce neuroinflammation, potentially slowing cognitive decline, while also improving lipid metabolism, lowering cholesterol absorption, and preventing age-related metabolic issues [113,114,115]. *Lactobacillus* and *Bifidobacterium*’s anti-inflammatory effects indirectly promote bone density and muscle function by enhancing nutrient absorption and reducing tissue breakdown [65,116,117]. Supplementing *Lactobacillus* and *Bifidobacterium* in older adults can ease conditions like constipation, irritable bowel syndrome (IBS), inflammatory bowel disease (IBD), and antibiotic-associated diarrhea while maintaining microbial diversity, which often decreases with age [118,119].

*Lactobacillus* and *Bifidobacterium* are well-known probiotics that may support healthy aging by improving gut microbiota, boosting immune responses, producing beneficial metabolites like SCFAs, and potentially managing inflammation and oxidative stress linked to aging [27]. However, using these probiotics in older adults poses challenges. Benefits depend on the strain, as their effects on immunity, gut health, and metabolism vary, making it hard to target aging-specific issues [120]. Their precise impact on aging markers like telomere shortening, mitochondrial dysfunction, or epigenetic changes remains unclear, as most studies are preclinical [121,122,123,124]. Colonization is often hindered by stomach acid, bile salts, and oxygen exposure, especially for anaerobic strains like *Bifidobacterium* [125]. Factors such as pH and temperature affect traits like surface hydrophobicity and mucosal adhesion, and many strains require continuous supplementation to stay in the gut [126]. Aging-related immune decline, gut microbiota shifts, chronic conditions, medications, and antibiotics further reduce their effectiveness [127]. Delivering enough viable bacteria is challenging, as stability varies in products like fermented foods, powders, and capsules, with some methods reducing bacterial viability during storage or digestion [128]. Multi-strain formulations require careful balancing due to interactions that may affect efficacy or metabolism [128]. Clinical evidence is limited; trials using *Lactobacillus rhamnosus* and *Bifidobacterium animalis* did not significantly reduce antibiotic use or improve systemic immunity in older adults [129]. Many studies are short-term, underpowered, or involve diverse elderly populations, making conclusions difficult. The obligate anaerobic nature of *Bifidobacterium* adds to its culturing challenges [130]. Despite this, *Lactobacillus* and *Bifidobacterium* show great potential for promoting healthy aging.

## 4. Mechanism of Action, Application, and Challenges of Utilizing *Lactobacillus* and *Bifidobacterium* in Autoimmune Disease Therapy

Autoimmune diseases occur when the immune system becomes overactive and mistakenly attacks the body’s own tissues [131]. Studies show that gut microbiota dysbiosis, an imbalance in the intestinal microbial community, is connected to the onset and progression of autoimmune conditions such as Amyotrophic lateral sclerosis (ALS), type 1 diabetes, rheumatoid arthritis, lupus, and multiple sclerosis [131]. Probiotics offer a promising way to manage gut microbiota, restoring balance by promoting beneficial microbes and outcompeting harmful ones [132]. Research in animals and humans indicates probiotics can delay or prevent autoimmune diabetes in non-obese diabetic (NOD) mice, lower inflammatory cytokines, and reduce joint damage in rheumatoid arthritis [104,133]. They also improve gut microbiota composition in conditions like systemic lupus and multiple sclerosis. However, results vary based on strain, disease, and study design [134,135]. Various probiotic strains hold promise for supporting patients with autoimmune diseases through their differentiation effects [136]. Probiotic formulations may involve single-strain or multispecies blends tailored to specific autoimmune conditions [137]. Creating probiotic formulations for autoimmune disease treatment involves selecting strains with known immunomodulatory benefits, ensuring they survive and thrive in the right locations, and tailoring them to the host and microbiome’s specific needs [138,139]. Next-generation probiotics (NGPs), developed through sequencing and bioinformatics, leverage synthetic biology and gene editing for targeted therapeutic applications [140]. These formulations often blend strains like *Lactobacillus* and *Bifidobacterium*, sometimes enhanced with technologies like nano-encapsulation or genetic engineering to improve their impact. While promising, current research indicates that probiotics are most effective as supplementary therapies rather than standalone treatments until more clinical studies are completed [138].

**Figure 4:**
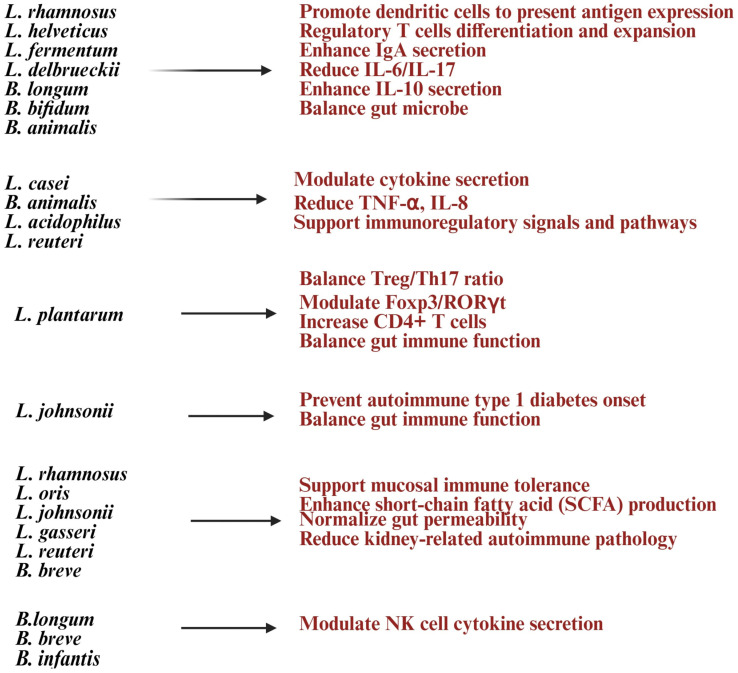
List of *Lactobacillus* and *Bifidobacterium* strains and their health benefits in autoimmune diseases. Created in https://BioRender.com (accessed on 3 October 2025).

*Lactobacillus* and *Bifidobacterium* help strengthen the gut barrier by improving tight junctions, reducing gut permeability (“leaky gut”), and lowering exposure to inflammatory triggers [141,142] (Figure 4). *Lactobacillus* species such as *Lactobacillus casei*, *Lactobacillus rhamnosus*, *Lactobacillus acidophilus*, and *Lactobacillus reuteri* are known for their immunomodulatory and barrier-strengthening properties [27]. *Lactobacillus* and *Bifidobacterium* probiotics play a significant role in immune regulation by influencing T and B lymphocyte activity [13]. They promote Treg expansion, enhance mucosal IgA production, and balance T-helper cells by increasing anti-inflammatory Tregs while reducing Th17/Th1 inflammation. These probiotics modulate cytokines, boosting IL-10 and TGF-β while reducing TNF-α and IL-6, to maintain a balance between pro- and anti-inflammatory responses [8,16,143]. SCFAs like butyrate, acetate, and propionate support Treg differentiation, suppress pro-inflammatory Th17 cells, and alter gene expression in immune cells through histone acetylation [8]. By fostering Treg expansion and anti-inflammatory cytokines, these probiotics may improve immune tolerance and reduce autoimmune conditions like rheumatoid arthritis, multiple sclerosis, and inflammatory bowel disease [99]. *Bifidobacterium* species such as *Bifidobacterium bifidum* and *Bifidobacterium animalis* support Treg induction and help lower pro-inflammatory responses [144]. Molecules from *Lactobacillus* (like lipoteichoic acid and peptidoglycan) and *Bifidobacterium* (like surface proteins and polysaccharides) interact with DCs to aid their maturation and antigen presentation, maintaining immune tolerance [145,146]. NK cell function is crucial for infection control and disease progression, and dysfunction in NK cells can impact the entire immune system [147,148]. Studies have shown that probiotic formulations with specific Gram-positive bacterial strains can enhance IL-10 levels in peripheral blood mononuclear cells (PBMCs) and NK cells, providing benefits for autoimmune diseases [149].

Clinical trials suggest that multi-strain probiotics containing *Lactobacillus* and *Bifidobacterium* can enhance gut barrier function, lower systemic inflammation, and improve tolerance to dietary antigens, offering relief for conditions like IBS and gut-related autoimmune disorders [117,150,151] (Figure 4). Research shows their anti-inflammatory effects and immune modulation may also help ease joint inflammation in rheumatoid arthritis (RA) [8]. These probiotics can work alongside immunosuppressive drugs, reduce side effects, and support immune balance during treatment [136]. They achieve these benefits by modulating gut bacteria, producing helpful metabolites, strengthening the gut lining, and interacting with immune cells [8,136] (Figure 4). This makes them promising, strain-specific options for restoring immune tolerance and managing chronic inflammation in autoimmune diseases.

Using *Lactobacillus* and *Bifidobacterium* to treat autoimmune diseases comes with several challenges [136]. While *Lactobacillus* is known for its health benefits, its role in autoimmune conditions is still unclear, reflecting the complexity of using probiotics for such treatments. These lactic acid bacteria have immunomodulatory properties and help balance gut microbiota, showing potential for conditions like type 1 diabetes, rheumatoid arthritis, lupus, Graves’ disease, and inflammatory bowel disease [152]. However, their effectiveness depends on the host’s individual background. Safety concerns, such as antibiotic resistance gene transfer, and the mechanisms of action, require thorough investigation [153,154]. *Lactobacillus reuteri* is beneficial for healthy individuals but might worsen autoimmune issues in predisposed hosts [136]. Host genetics, like HLA types, play a role in whether probiotics improve or exacerbate autoimmune responses, adding to the unpredictability [155]. Environmental factors like diet, antibiotics, stress, and geography further influence probiotic effectiveness by impacting gut microbiota and barrier integrity [156]. Additionally, a leaky gut in autoimmune-prone individuals raises the risk of probiotics escaping the gut [157]. Rare infections linked to some *Lactobacillus* strains (e.g., *Lactobacillus rhamnosus*, *Lactobacillus paracasei*) and *Bifidobacterium* species (e.g., *Bifidobacterium longum*, *Bifidobacterium breve*) include bacteremia, endocarditis, urinary tract infections, and neonatal sepsis in immunocompromised individuals [158,159,160]. While animal and in vitro models show promise, especially with pathways like JAK/STAT and NF-κB, human immune interactions remain complex and not fully understood [161]. Probiotics and their metabolites may also struggle to reach effective concentrations at target tissues due to metabolic competition, mucosal barriers, or rapid clearance, although techniques like microencapsulation and nano-delivery could help address these issues [162]. Autoimmune patients need personalized approaches since genetic, microbiome, and environmental differences make one-size-fits-all solutions ineffective [163]. The variability in probiotic dosage, strain formulations, and combinations makes it hard to replicate findings across studies [162].

## 5. Mechanism of Action, Application, and Challenges of Utilizing *Lactobacillus* and *Bifidobacterium* in Neurodegenerative Disorders

Neurodegenerative diseases such as Alzheimer’s, Parkinson’s, Amyotrophic lateral sclerosis (ALS), and Huntington’s are progressive conditions with complex origins, including protein buildup, oxidative stress, neuroinflammation, mitochondrial dysfunction, and immune system issues [164,165]. The gut microbiome significantly influences these diseases via the microbiota–gut–brain axis, impacting neuroinflammation, gut barrier integrity, immune responses, and neurotransmitter production [166,167,168]. Dysbiosis, or an imbalance in gut bacteria, is often observed in these disorders [138,169]. It includes an increased presence of pro-inflammatory strains like *Streptococcus*, *Alistipes*, *Ruminococcus*, *Enterococcus*, and *Desulfovibrio*, alongside a reduction in beneficial butyrate-producing bacteria such as *Faecalibacterium*, *Lachnospira*, *Roseburia*, *Blautia*, and *Prevotella* [138,169]. Probiotics provide therapeutic benefits by replenishing beneficial bacteria, boosting neuroprotective metabolites like butyrate, and offering anti-inflammatory and antioxidant effects [97,166].

**Figure 5:**
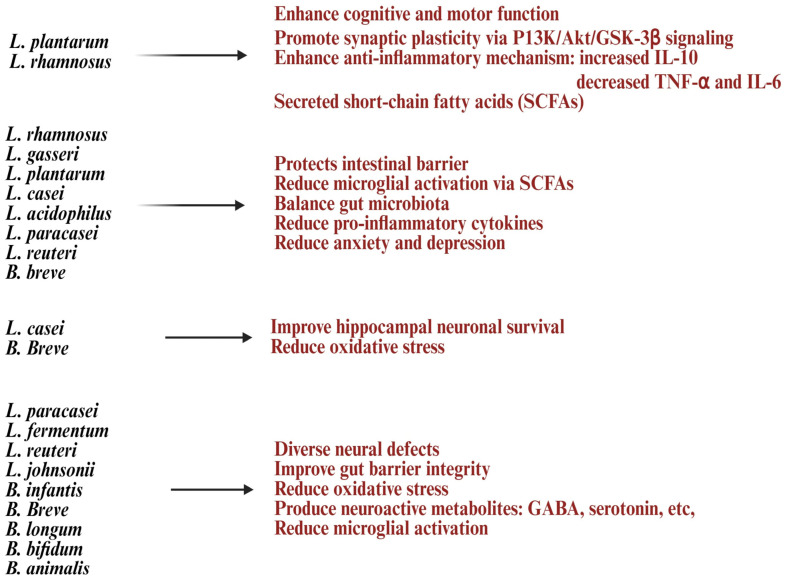
List of *Lactobacillus* and *Bifidobacterium* strains and their health benefits in neurodegenerative disorders. Created in https://BioRender.com (accessed on 3 October 2025).

*Lactobacillus* and *Bifidobacterium* are well-known probiotics that play a key role in the gut–brain axis (GBA), a complex system connecting the gastrointestinal (GI) tract, immune system, and central nervous system (CNS) through neural, hormonal, and immune pathways [13] (Figure 5). *Lactobacillus* and *Bifidobacterium* also influence neurotrophic factors and neurotransmitter pathways like brain-derived neurotrophic factor (BDNF), serotonin, and gamma-aminobutyric acid (GABA), and maintain microglial balance [108,170,171]. They help reduce pro-inflammatory pathways, enhance vagus nerve signaling for better brain communication and emotional regulation, and combat oxidative stress with antioxidant defenses, which is crucial for neurodegenerative disorders [172,173]. Furthermore, they may prevent the accumulation of harmful protein aggregates [4]. These probiotics support the intestinal barrier by boosting mucin production, tight-junction proteins like occludin and claudin-1, and reducing intestinal permeability, which helps block neuroinflammatory mediators from entering the bloodstream [174]. This protective barrier limits exposure to bacterial lipopolysaccharides (LPS) and pro-inflammatory cytokines, which can trigger CNS inflammation [174,175]. *Lactobacillus* and *Bifidobacterium* secreted compounds like SCFAs, bacteriocins, and hydrogen peroxide that lower gut pH, inhibit harmful bacteria, and indirectly support CNS health by reducing systemic inflammation [174,176]. By interacting with DCs and T cells, these probiotics encourage regulatory T cell (Treg) differentiation, balance Th1/Th2 immune responses, increase anti-inflammatory cytokines like IL-10 and secretory IgA, and reduce pro-inflammatory cytokines (e.g., IL-1β, IL-18, TNF-α) in the hippocampus and other brain regions [177,178]. These actions help regulate CNS microglial activity, preventing neuronal damage caused by chronic inflammation [177] (Figure 5).

Combining probiotic strains like *Lactobacillus* and *Bifidobacterium* species can work together to improve motor and cognitive symptoms in Parkinson’s disease models [151,179]. Taking *Lactobacillus casei* and *Bifidobacterium breve* orally has been shown to enhance short-term memory by improving learning performance and antioxidant capacity in the demyelinated corpus callosum, highlighting a potential therapeutic approach for multiple sclerosis and other neurodegenerative disorders [180]. These strains increase levels of α-Klotho, Sirtuin1, HO-1, and Nrf2 (these pathways play a crucial role in neuroprotection) while reducing pro-inflammatory genes like IL-1β and IL-18 in the hippocampus. They also boost BDNF expression, protect hippocampal neurons, and reduce neurodegeneration [180]. They also show potential in treating depression, with both clinical and preclinical studies providing encouraging results [181]. Probiotics offer neuroprotective benefits by strengthening the gut barrier, regulating immunity, producing neuroactive compounds, and lowering oxidative stress. Multi-strain combinations are particularly effective, making them a promising addition to treatments for neurodegeneration and cognitive decline. Probiotic formulations should aim to balance the gut microbiome, increase butyrate-producing bacteria, and reduce inflammation.

*Lactobacillus* and *Bifidobacterium* are gaining attention as potential treatments for neurodegenerative disorders like Alzheimer’s, Parkinson’s, Huntington’s, multiple sclerosis, and ALS due to their effects on the microbiota–gut–brain axis. They can reduce neuroinflammation, produce neuroactive compounds like GABA and serotonin, and enhance neuronal resilience. However, challenges remain. Not all strains provide neuroprotection; for instance, *Lactobacillus casei* and *Bifidobacterium breve* perform well together in aging mouse models, but monotherapy might be less effective [182,183]. Variations in metabolic activity and neurotransmitter production make standardizing treatments difficult. Oral probiotics face survival issues in the gastrointestinal tract due to stomach acid, bile salts, and enzymes. Gut microbiota composition and colonization capacity also vary, impacting outcomes. Factors like biofilm formation and gut lining adherence, unique to each strain, influence persistence and efficacy. Advanced delivery methods like microencapsulation improve stability but face hurdles like polymer compatibility, storage, and regulatory challenges. Manufacturing, storage, and transport conditions also affect effectiveness. Personalized responses based on diet, age, genetics, medications, and diseases add complexity, as some individuals may see no benefit. While generally considered safe, risks exist. Future efforts should focus on personalized regimens, advanced targeting systems like nano-armor probiotics, and larger clinical trials.

## 6. Clinical Outcomes That Align with the Findings from the Preclinical Studies

Early clinical trials confirm preclinical findings, showing that *Lactobacillus* and *Bifidobacterium* strains restore intestinal microbiota diversity disrupted by chemotherapy or radiotherapy, reducing gastrointestinal issues like diarrhea and mucositis [184,185]. These probiotics enhance the immune system, with strains like *Lactobacillus rhamnosus* and *Bifidobacterium* boosting markers such as NK cell activity and anti-inflammatory cytokines [116]. In older adults, they support intestinal health, reduce systemic low-grade inflammation through beneficial fermentation, and increase SCFA production [186,187]. For autoimmune conditions like ulcerative colitis and rheumatoid arthritis, probiotics help modulate microbiome-immune interactions, shifting the Th17/Treg balance toward regulatory phenotypes and lowering inflammation markers [188]. They also improve bloating and stool regularity in autoimmune gut disorders, influence microbiota-derived metabolites, and reduce inflammatory markers linked to neuroinflammation [189]. Small clinical studies consistently report improved MMSE scores and reduced depression/anxiety symptoms [190].

## 7. Research Gaps Contributing to Inconsistencies Between Clinical Outcomes and the Findings from the Preclinical Studies

Research on probiotics like *Lactobacillus* and *Bifidobacterium* reveals significant gaps between animal studies and human trials due to biological differences, study designs, and translational challenges [27,191]. Preclinical studies indicate that *Lactobacillus* may support gut barrier protection, immune system modulation, and metabolism. Human trials partially back these findings, with results varying by strain, host, and environment [27,191,192]. Animal models like rodent colitis or germ-free mice provide insights into mechanisms but do not account for human genetic diversity, microbiota differences, or diet-related interactions [192,193] (Table 1). Preclinical studies often use probiotic dosages and durations impractical for humans [194]. This suggests mechanisms like barrier reinforcement, SCFA signaling, and immune modulation, but rarely predict the scale or variability of human responses [195]. Human outcomes are typically less pronounced, more variable, and highly context-dependent compared to preclinical findings [104,190]. Translational outcomes depend on factors like baseline microbiota, genetics (e.g., FUT2 genotype), diet, co-administered therapies, dosage, and treatment duration. Strains like *Lactobacillus plantarum*, *Lactobacillus rhamnosus*, and *Lactobacillus reuteri* show promise in mice, but human trials often produce inconsistent results due to factors like dosage, age, and maternal supplementation timing [196]. For instance, *Lactobacillus rhamnosus* reduces allergy and eczema in mice, but human trials yield mixed results [197,198]. Similarly, *Lactobacillus plantarum* improves lipid profiles in rodents but only modestly reduces cholesterol (5–10%) in humans, with significant variability [199]. While *Lactobacillus* enhances tight junctions and reduces endotoxemia in mice, its effects in humans are minimal, with biomarkers like zonulin showing inconsistent responses [200,201]. Compounds deemed safe in mice may still cause gastrointestinal or immune reactions in humans, underscoring the need for cautious translation [201].

Research gaps include the lack of standardized outcome measures, like patient-reported versus biomarker-based, and core outcome sets (COS) across diseases, which complicate meta-analyses and evidence synthesis [202]. Strain-specific effects are often inconsistently reported, with many studies relying on combination preparations or poorly characterized formulations [139]. The mechanisms connecting probiotic use to systemic immunomodulation, anti-cancer effects, and neuroprotection remain unclear [13]. More investigation is needed into the gut–brain and gut-immune axes, especially in long-term disease models [13]. Clinical trials frequently lack large-scale, multicenter randomized clinical trials with extended follow-ups, and variability in patient populations and baseline microbiota reduces reproducibility [203]. Subgroup analyses by age, disease type, diet, and genetics are also limited. There is a lack of regulatory guidance on dosing, duration, and formulation, while safety monitoring in immunocompromised individuals and the frail elderly is underexplored. Additionally, limited studies have looked at how probiotics interact—whether synergistically or antagonistically—with chemotherapy, immunotherapies, or anti-inflammatory drugs. We have listed the most common factors interfering with consistent outcomes for probiotic use in humans, which do not align with preclinical outcomes (Table 1).

To maximize the benefits of *Lactobacillus* and *Bifidobacterium*, combining mechanistic data with well-structured, stratified clinical trials is key [190,204]. Focusing on disease-specific outcomes for probiotics will standardize reporting. Large placebo-controlled trials should account for baseline microbiota, age, and comorbidities. Advanced methods like metagenomics and metabolomics can reveal pathways, while cohort studies should link probiotics to outcomes and quality of life, particularly in cancer, aging, autoimmune, and neurodegenerative conditions. Future research should adopt precision nutrition strategies, factoring in baseline microbiota, genetics, and diet to refine probiotics [190,205]. Longitudinal randomized clinical trials stratified by microbiota, genetics, and clinical subtypes are essential [206]. Multi-omics tools can connect mechanistic insights to outcomes, while postbiotics and metabolites might act as biomarkers bridging preclinical and clinical data [207]. Ensuring safety and optimizing formulations are crucial for vulnerable groups like the elderly or immunocompromised.

## 8. Conclusions

*Lactobacillus* and *Bifidobacterium* probiotics are well-known for their benefits on gut health, with recent studies showcasing their broader role in health and disease management. They help restore immune function in aging and conditions like cancer, autoimmune, and neurodegenerative disorders. As an adjuvant cancer therapy, they support immune balance, balance gut microbiota, and improve intestinal function, which results in minimizing the adverse effects of chemotherapy, radiotherapy, and checkpoint inhibitors. Research indicates these probiotics activate and expand anti-cancer immune cells like NK and CD8+ T cells, enhancing their cytotoxic activity, IFN-γ secretion, and recruitment to tumors. However, challenges include strain-specific effects, infection risks, inconsistent metabolite production, and lack of standardized clinical protocols. Future research should focus on personalized microbiome profiling, optimized strains and dosages, and innovative delivery systems like microencapsulation. Combining probiotics with genetic engineering or metabolite supplementation could lead to safer, more effective treatments, but thorough clinical trials are essential. Advancing these techniques and understanding molecular mechanisms is key to unlocking probiotics’ therapeutic potential and improving health outcomes.

## Figures and Tables

**Table 1:** Factors influencing differences in preclinical and clinical results of *Lactobacillus* and *Bifidobacterium* applications.

Factors	Preclinical Studies	Clinical Studies
Gut Microbiota/ physiology/immune system	It can be regulated. Probiotics might temporarily colonize mice and significantly influence immune markers or gut barrier function because of their simplified microbiota.	Humans have highly diverse microbiomes and immune adaptations that influence the survival, activity, and metabolic effects of *Lactobacillus*. Probiotic colonization tends to be temporary, with metabolic or immunomodulatory impacts often being weaker and highly personalized.
Diet/lifestyle	Diet and lifestyle can be adjusted or personalized.	Variable dietary factors, medications, and previous microbiome exposures contribute to confounding factors.
Genetic and environmental factors	Genetically uniform strains are common, and environmental conditions can be managed.	Substantial genetic heterogeneity makes it challenging to manage due to population variability.
Duration	Short-term interventions and acute effects	Long-term chronic effects and safety assessments are not as frequently studied.
Sample size	Can reach 100 or more, and it is easy to scale up.	Often ranging from 10 to 1000, it is not always easy to scale up.
Mechanism	SCFAs increased acetate and propionate levels, providing barrier protection.	Outcomes depend on diet and transient colonization.
	Boosted Treg cells while reducing pro-inflammatory cytokines.	Small reductions in inflammation markers with subject variability.
	In colitis models, SCFAs enhanced junction integrity by increasing ZO-1, occludin, and claudin-1.	Some improvements in mucosal barrier proteins have been noted, though functional outcomes are modest.
	mitigated stress and anxiety through the vagus-OXT axis.	Evidence is limited, but perinatal mood benefits have been observed in pilot RCTs.
	Consistent anti-inflammatory responses, enhanced mucosal IgA production, or regulated cytokine activity.	Variable effects, occasionally showing statistical significance in specific subpopulations, but often not reproducible across different studies.
Endpoint measurements	Invasive sampling, such as from tissue or the intestinal lumen, provides detailed mechanistic insights	Non-invasive methods, such as stool samples and blood biomarkers, offer insights but limit mechanistic resolution.

## Abbreviations

The following abbreviations are used in this manuscript:

TLR	Toll-like receptor
Tregs	Regulatory T cells
Th	T helper
IL	Interleukin
TGF-β	Transforming growth factor-beta
IgA	Immunoglobulin A
DCs	Dendritic cells
SCFA	Short-chain fatty acids
MyD88	Myeloid differentiation primary response 88
DNA	Deoxyribonucleic acid
NK	Natural killer
CD8	Cluster of differentiation 8
IFN-γ	Interferon-gamma
iNOS	Inducible nitric oxide synthase
NO	Nitric oxide
NKG2D	Natural killer group 2, member D
CCR7	C-C chemokine receptor type 7
PD-1	Programmed death protein-1
CRP	C-reactive protein
GABA	Gamma-aminobutyric acid
IBS	Irritable bowel syndrome
IBD	Inflammatory bowel disease
RA	Rheumatoid arthritis
ALS	Amyotrophic lateral sclerosis
GBA	Gut–brain axis
CNS	Central nervous system
BDNF	Brain-derived neurotrophic factor
